# A critical assessment of dose effects of post‐thaw CD34 on autologous stem cell transplantation treatment of haematological malignancies

**DOI:** 10.1002/jha2.665

**Published:** 2023-03-02

**Authors:** Gustavo de Carvalho Duarte, Andrew Butler, Gavin Atkinson, Krishna Badami, Wen‐Hua Wei

**Affiliations:** ^1^ New Zealand Blood Service Christchurch New Zealand; ^2^ Haematology Department Christchurch Hospital Christchurch New Zealand; ^3^ Centre for Biostatistics, Division of Population Health, Health Services Research and Primary Care The University of Manchester Manchester UK

**Keywords:** CD34, clinical outcome, cryopreservation, haematological malignancy, viability

## Abstract

A consensus threshold of pre‐cryopreservation CD34‐positive cells (CD34s) has been used as the minimum dose to initiate autologous stem cell transplantation (ASCT). Advances in cryopreservation posed a debate whether post‐thaw CD34s might be a superior surrogate instead. We addressed the debate in this retrospective study of 217 adult ASCTs in five different haematological malignancies treated at a single centre. We showed that post‐thaw CD34s was highly correlated with pre‐cryopreservation CD34s (*r* = 0.97) and explained ∼2.2% (*p* = 0.003) of the variation of the post‐thaw total nucleated cell viability that however had no power to predict engraftment outcomes. After stratifying the ASCT cases into four dose groups based on post‐thaw CD34s reinfused, stepwise multivariate regression analyses detected significant effects in dose group and interactions with diseases for neutrophil and platelet recovery respectively. The significant dose effects and interactions were triggered by two technical outliers in the low dose group, and disappeared in the repeated regressions after exclusion of the outliers where disease and age were the significant predictors remained. Our data clearly support the validity of the consensus threshold in ASCT applications but also highlight neglected conditions where monitoring post‐thaw CD34s and clinical attributes are valuable.

## 1 INTRODUCTION

Autologous hematopoietic stem cell transplantation (ASCT) after high‐dose chemotherapy has been shown to extend survival compared with less intensive treatment for haematological malignancies, such as multiple myeloma (MM) and lymphomas. In transplant‐eligible patients with myeloma first‐line treatment with high dose melphalan and ASCT is associated with a higher probability of complete remission, improved progression free‐survival and overall survival [1, 2]. Similarly, efficacy has been demonstrated in the treatment of chemosensitive relapsed lymphomas [3, 4, 5, 6].

A successful ASCT relies on successes in stem cell collection, cryopreservation, myeloablative chemotherapy and stem cell re‐infusion. A number of variables within these procedures could ultimately impact clinical outcomes [7]. The number of CD34+ positive cells (CD34s) infused has been considered to be the most important factor associated with haematopoietic reconstitution following myeloablative treatment [1, 8, 9, 10, 11, 12, 13]. Administration dose of CD34s under 1.5–2.5 × 10^6^ per kilogram of recipient body weight (cells/kg) was previously reported to lead to delayed neutrophils and platelets engraftment [1]. The threshold of 2.0 × 10^6^ CD34 cells/kg harvested before cryopreservation, has been established as the minimum dose to ensure successful neutrophil and platelet recovery [1, 14]. On the other hand, cryopreservation techniques are widely used in the clinical settings of ASCT to maintain essential regenerative properties for stem cells. Transplant centres are required to routinely measure CD34 recovery and viability in a post‐thaw sample as indicated in the current FACT‐JACIE International standards stating ‘For cellular therapy products undergoing manipulation that alters the final cell population, a relevant and validated assay, where available, shall be employed for evaluation of the viable target cell population before and after the processing procedures’ [15]. However, the clinical significance of cell loss and the relationship between post‐thaw CD34 and engraftment count recovery is uncertain.

A number of variables during the process of cryopreservation could influence quality and quantity of viable CD34s mostly concerned for re‐infusion, including total nucleated cell (TNC) concentration, white cell count (WCC), cryoprotectant agent, conditions of the freezing process, temperature and duration of storage [7, 16]. There have been concerns over cell losses during cryopreservation and thawing that might jeopardise haematopoietic reconstitution and engraftment. The enumeration of CD34s post‐cryopreservation and prior to reinfusion could therefore be a clinically important surrogate to predict graft success or failure [9]. Several reports suggest that the post‐thaw CD34 number is a more reliable predictor of engraftment than the pre‐cryopreservation counterpart, especially for platelets [11, 12, 16, 17]. However, these studies used small numbers of patients often heterogeneous in characteristics such as age and ethnicity [10, 11, 12, 17], with limited power to reliably extrapolate different aspects of patient treatment outcomes, or to provide clear recommendations on the minimum safe dose of post‐thaw CD34s similar to the aforementioned minimum dose of harvest CD34s [1, 8, 10].

To mitigate the issues of heterogeneity and sampling, this study used a large population of patients receiving ASCT at a single centre. We first evaluated attributes associated with collection, cryopreservation and thawing processes and their impact on the graft. We then investigated impact of dose of post‐thaw CD34s on post‐ASCT neutrophil and platelet kinetics in conjunction with patient characteristics.

## 2 MATERIALS AND METHODS

### 2.1 Patients

This is a retrospective study of patients undergoing ASCT at Christchurch Hospital, New Zealand, between January 2017 and May 2022. All patients were over 18 years, had stem cell mobilisation, collection, ASCT, and clinical monitoring completed at the same centre during the study period. This is a minimal risk observational study which did not require ethical approval nor written informed consent, according to the Standard Operating Procedures for the Health and Disability Ethics Committees of New Zealand. The study was approved by the Clinical Advisory Group (i.e., institutional review board) the New Zealand Blood Service.

### 2.2 Stem cell mobilization, harvest and processing

Peripheral blood stem cells (PBSC) were mobilized with granulocyte colony‐stimulating factor (G‐CSF) with or without chemotherapy according to disease characteristics. Leukapheresis was performed using automated system apheresis equipment (Spectra Optia, Terumo BCT, Lakewood, CO, USA). Products were processed on the same day (<4 h) after harvest or stored overnight at 4°C and processed the following morning. All the products were processed in less than 24 h after harvest. After centrifugation and plasma volume reduction, PBSC were diluted 1:2 with DMSO 10% and autologous plasma. All PBSC products were frozen in a controlled‐rate freezer and stored in −150°C Nitrogen tanks. Reference samples were prepared and cryopreserved with the main unit. CD34 enumeration and viability were assessed at the time of harvest (pre‐freezing) and 5 to 7 days after being cryopreserved.

### 2.3 Conditioning, stem‐cell reinfusion and follow‐up

The conditioning used is summarized in Table S1. The products were thawed at the bedside using a water bath at 37°C immediately prior to re‐infusion. CD34 enumeration and viability were assessed using multi‐parameter flow cytometry according to ISHAGE protocol [10].

After re‐infusion, patients were observed for 4 h and discharged the same day for follow‐up in the outpatient department. Post‐transplant G‐CSF was not mandated by protocol but could be given at the treating physician's choice, for example, for severe sepsis. Neutrophil recovery was defined as the first of three consecutive days’ post‐re‐infusion with an absolute neutrophil count higher than 0.5 × 10^9^/L (https://www.cibmtr.org/manuals/fim/1/en/topic/q8‐11‐initial‐anc‐recovery) and the number of days taken to achieve the point was recorded as ANC05. Platelet recovery was defined as the first of three consecutive days where the platelet count was greater than 20 × 10^9^/L without platelet transfusion support in the preceding 7 days (https://www.cibmtr.org/manuals/fim/1/en/topic/q12‐14‐initial‐platelet‐recovery) and the number of days taken to achieve the point was recorded as Platelet20. Platelets were continuously monitored until a count higher than 50 × 10^9^/L was achieved and the number of days taken to achieve the point was recorded as Platelet50.

### 2.4 Data collection and analysis

Patient demographic data including sex and age at transplantation, together with number of stem cell collections and overnight storage were obtained from the hospital's electronic records. A total of 217 patients with a total of 347 collections each with at least 2.0 × 10^6^ harvest CD34 cells/kg were included in the study (Tables 1 and 2).

**TABLE 1:** Summary information of autologous stem cell transplantation (ASCT) cases and the leukapheresis product characteristics.^*^

	All	Amyloidosis	Hodgkin Lymphoma	B‐cell NHL	T‐cell NHL	Multiple Myeloma
Total patients	217	4	11	59	8	135
Median age (years)	59	64	41	58	60	61
Sex						
Male	137 (63.1%)	3 (75.0%)	8 (72.7%)	45 (76.3%)	4 (50.0%)	77 (57.0%)
Female	80 (36.9%)	1 (25.0%)	3 (27.3%)	14 (23.7%)	4 (50.0%)	58 (43.0%)
Collection	347	5	21	98	10	213
Collection/patient	1.6	1.25	1.9	1.7	1.25	1.6
Overnight storage						
Yes	101	1	5	26	4	65
No	243	4	15	72	6	146
Harvest volume (mL)	235.5 ± 48.0	200.4 ± 53.6	237.1 ± 51.4	242.3 ± 48.3	217.6 ± 52.2	234.0 ± 46.9
WCC (10^9^/L)	186.8 ± 94.2	307.7 ± 143.5	199.3 ± 92.0	211.9 ± 92.1	222.2 ± 128.2	169.6 ± 88.0
TNC (10^8^/kg)	5.0 ± 3.0	7.1 ± 2.8	5.4 ± 4.2	5.8 ± 2.8	6.3 ± 4.7	4.5 ± 2.6
Harvest CD34 (10^6^ cells/kg)	3.7 ± 3.4	3.4 ± 2.0	2.7 ± 2.4	3.4 ± 4.3	5.1 ± 4.0	3.9 ± 3.0
Post‐thaw CD34 (10^6^ cells/kg)	2.8 ± 2.6	3.0 ± 1.7	2.1 ± 1.7	2.5 ± 3.1	3.5 ± 1.9	2.9 ± 2.4
Post‐thaw CD34 viability (per unit)	75.6% ± 12.9%	89.2% ± 4.8%	78.8% ± 9.8%	74.8% ± 12.7%	76.1% ± 11.5%	75.2% ± 13.2%
Post‐thaw TNC viability (per unit)	63.6% ± 13.9%	58.8% ± 11.4%	67.6% ± 14.5%	63.0% ± 14.8%	67.1% ± 13.4%	63.4% ± 13.5%

* Three collections with no information about overnight storage; quantitative measures were presented in the form of mean ± standard deviation.

**TABLE 2:** Summary information of autologous stem cell transplantation (ASCT) cases and treatment characteristics.^*^

	All	Amyloidosis	Hodgkin Lymphoma	B‐cell NHL	T‐cell NHL	Multiple Myeloma
Pre‐freeze CD34 (10^6^ cells/kg)	4.80 ± 2.36	4.25 ± 1.68	5.11 ± 2.22	5.40 ± 3.47	6.35 ± 3.82	4.43 ± 1.48
Post‐thaw CD34 (10^6^ cells/kg)	3.56 ± 1.62	3.75 ± 1.42	3.92 ± 1.55	3.92 ± 2.29	4.41 ± 1.53	3.31 ± 1.20
Case grouping* (n)	217	4	11	59	8	135
G1 (≤ 2 × 10^6^/kg)	20	0	1	4	0	15
G2 (2 ‐ 3 × 10^6^/kg)	79	1	4	20	2	52
G3 (3 ‐ 4 × 10^6^/kg)	59	2	1	18	2	36
G4 (> 4 × 10^6^/kg)	59	1	5	17	4	32
Post‐thaw TNC viability (per transplant)	63.1% ± 13.5%	59.0% ± 13.1%	67.0% ± 13.8%	61.7% ± 15.3%	69.6% ± 12.1%	63.2% ± 12.7%
Patients achieved ANC05 (*n*)	215	4	11	57	8	135
Days to achieve ANC05	15.4 ± 5.9	13.5 ± 0.6	12.8 ± 2.5	14.6 ± 7.1	13.0 ± 3.1	16.2 ± 5.6
Patients achieved Platelet20 (*n*)	207	4	11	57	8	127
Days to achieve Platelet20	18.5 ± 5.1	22.5 ± 7.0	18.6 ± 6.4	19.8 ± 6.9	17.1 ± 2.6	17.8 ± 3.7
Patients achieved Platelet50 (*n*)	215	4	11	57	8	135
Days to achieve Platelet50	22.8 ± 13.4	24.3 ± 8.6	28.7 ± 30.3	27.2 ± 19.4	19.5 ± 6.1	20.6 ± 6.4

* Post‐thaw CD34 dose; for data analysis purposes, multiple collections per case (if any) were combined prior to cryopreservation; two cases died during the study period, neither achieved ANC05 but one achieved both Platelet20 and Platelet50; 10 cases with platelet levels never below Platelet20; 1 case with platelet level never below Platelet50; the mean and standard deviation of Post‐thaw CD34 levels were 1.80 ± 0.18, 2.59 ± 0.29, 3.48 ± 0.29, 5.51 ± 1.83 for G1 to G4, respectively.

**FIGURE 1:**
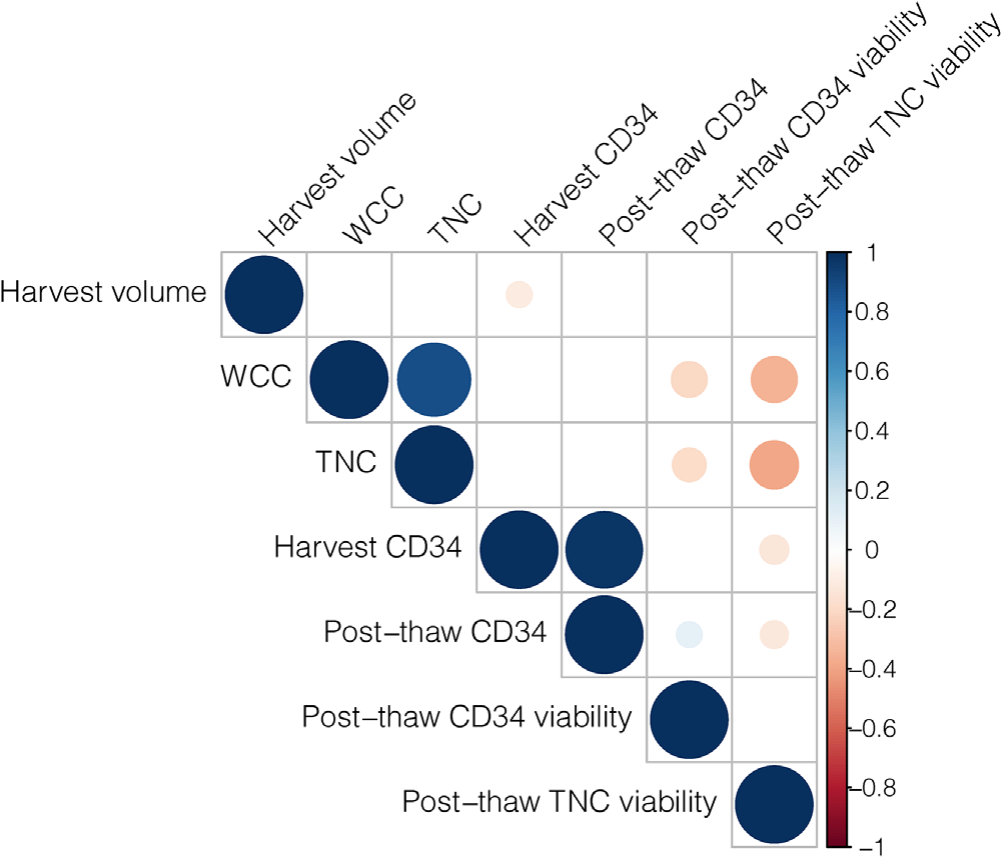
Pearson's pairwise correlations between measures for stem cell processing. Each correlation coefficient is represented by a colored pie where the size and color are indicated by the color panel on the right showing a positive correlation in blue and a negative correlation in red; on the diagonal are the correlation with self for each measure, that is, the correlation coefficient is 1 and thus in the full pie size with dark blue color; only significant correlations are plotted, and insignificant correlations are set blank.

Statistical analyses were performed using R [18] (v4.1.1) and R packages *MASS*, *stats*, *Hmisc*, *DescTools*, *ggpubr*, *corrplot* and *ggplot2*. Firstly, PBSC processing data were analysed as follows. Pearson's correlations were computed using the *rcorr()* function with p‐values calculated based on two‐tailed t tests, which were fed into the *corrplot()* function to generate a correlation heatmap (Figure 1). Considering post‐thaw total viability per collection as the outcome variable, a three‐step approach was taken to perform multivariable linear regression analyses using the *lm()* function and the *stepAIC()* function: (1) fitting the NULL model where all variables of interest without any interactions were fitted in a linear regression model; (2) fitting the FULL model where all variables of interest and their all possible interactions were fitted in a linear regression model; (3) the results of the NULL and the FULL models were fitted into the *stepAIC()* function with the direction parameter set as ‘both’ to perform a stepwise selection of the best model explaining the most phenotypic variance. The resultant best model concerns only covariates (i.e., variables and/or their interactions) with significant effects in the regression and was further analysed using the *anova()* function to quantify variance explained by each covariate and to examine significance using the built‐in F test.

Secondly, patients were grouped based on the dose of post‐thaw CD34s (D, in 10^6^ cells/kg): D ≤ 2 (G1), 2 < D ≤ 3 (G2), 3 < D ≤ 4 (G3), D > 4 (G4). Considering ANC05, Platelet20 and Platelet50 each as a treatment outcome variable respectively, (1) patients with neutrophil or platelet nadir above the outcome endpoint or not achieving the outcome endpoint were excluded (Table 2); (2) by dose group and diagnosis respectively, the cumulative percentages of patients were calculated as the number of patients who achieved the endpoint on each day divided by the total number of patients; (3) the three‐step multivariable linear regression analyses described above were performed to examine different combinations of variables including sex, dose group, diagnosis, age, harvest CD34s and total cell viability, and the final model fitted with only covariates with significant effects was reported.

## 3 RESULTS

Table 1 summarized 217 ASCTs and a total of 347 collections of PBSCs performed at Christchurch Hospital between January 2017 and May 2022. The indications for ASCTs were: MM (62.2%) B‐cell non‐Hodgkin lymphoma (NHL) (27.2%), Hodgkin lymphoma (5.1%), T‐cell NHL (3.7%) and amyloidosis (1.8%). Patients had a male preponderance, with a median age of 60 years and on average 1.6 collections. The average post‐thaw viabilities were 75.6% ± 12.9% for CD34 and 63.6% ± 13.9% for total nucleated cells (Table 1).

Highly significant positive correlations were observed between harvest CD34 and post‐thaw CD34 (Pearson's correlation coefficient *r* = 0.97, *p* = 3.4e‐223) and between WCC and TNC (*r* = 0.88, *p* = 5.1e‐112), suggesting these highly correlated measures could be used interchangeably (Figure 1). Post‐thaw CD34 was also positively correlated with post‐thaw CD34 viability (*r* = 0.11, *p* = 0.049). However, WCC and TNC were both negatively correlated with post‐thaw CD34 viability (*r* = −0.21 and *p* = 9.3e‐05, *r* = −0.18 and *p* = 5.8e‐04, respectively) and had even stronger negative correlations with post‐thaw TNC viability (*r* = −0.35 and *p* = 3.4e‐11, *r* = −0.38 and *p* = 1.3e‐13, respectively) that also negatively correlated with either harvest (*r* = −0.14 and *p* = 0.01) or post‐thaw CD34 (*r* = −0.12 and *p* = 0.02). These negative correlations, together with another marginally significant negative correlation between harvest volume and harvest CD34 (*r* = −0.11, *p* = 0.044), suggesting high cell (including CD34) counts were likely associated with reduced cell viabilities in the ASCT cases.

Considering post‐thaw TNC viability as the outcome variable for processing, linear regression analyses of the other variables listed in Table 1 identified three significant predictors: TNC (*p* = 1.1e‐13), post‐thaw CD34 (*p* = 0.003) and sex (*p* = 0.037), which jointly explained 16.5% of the total variance. Nevertheless, sex was highly significant (*p* = 8.6e‐14) when regressing to harvest volume explaining ∼15% of the total variation.

**FIGURE 2:**
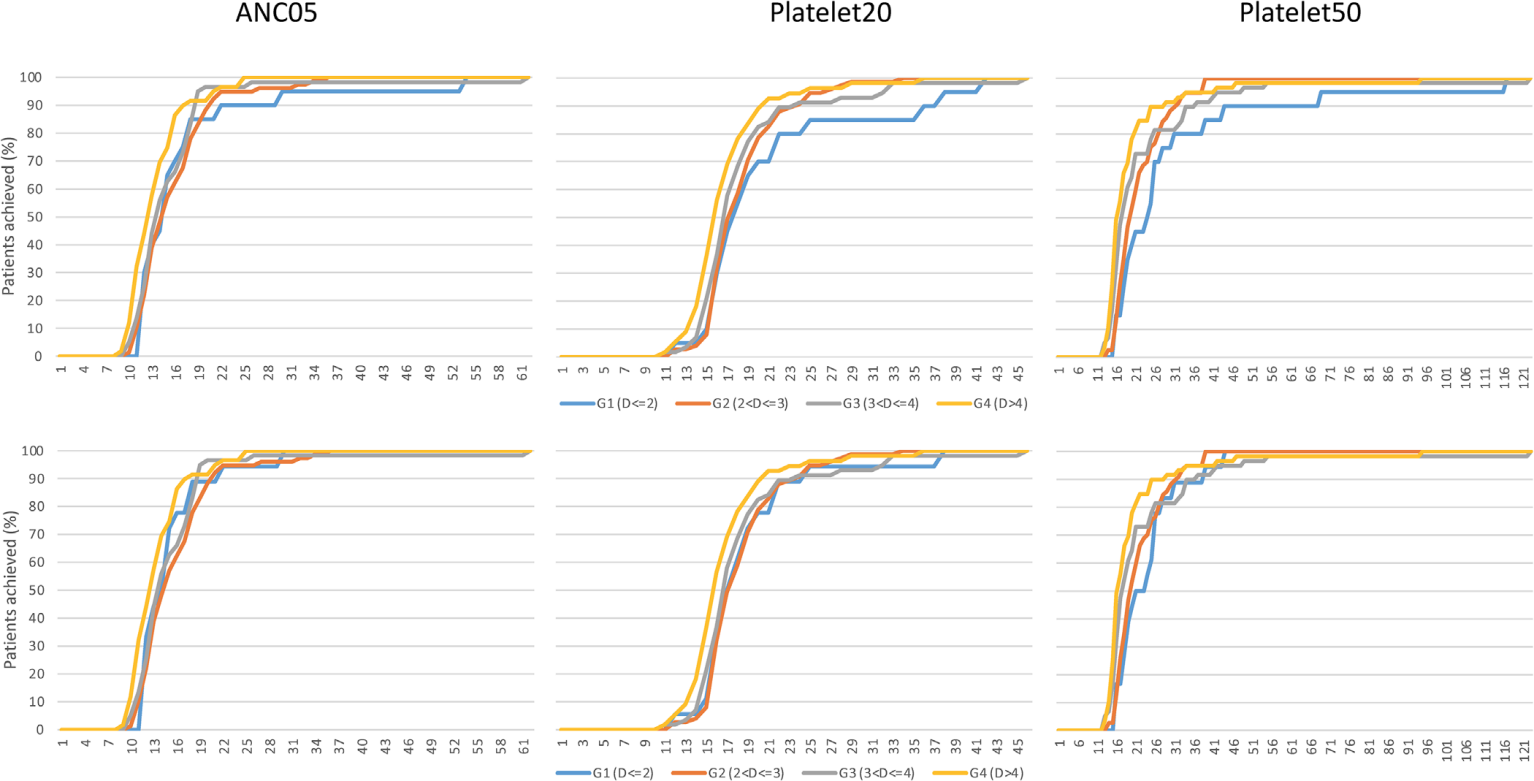
Cumulative percentage of cases achieved ANC05 (left column), Platelet20 (middle column) and Platelet50 (right column) endpoints by post‐thaw CD34 dose group. Top panel: concerning all autologous stem cell transplantation (ASCT) cases; bottom panel: concerning ASCT cases without two technical outliers identified in the low dose group.

Post‐thaw CD34 dose effects on treatment outcomes were explicitly examined after stratifying the 217 ASCT cases into four dose groups: 20, 79, 59 and 59 patients in G1 to G4, respectively (Table 2). Considering the curves of the cumulative percentages of cases that achieved the treatment endpoint of either ANC05, or Platelet20 or Platelet50, the G1 group was the slowest to recover among the four groups (Figure 2, top panel). Regression analyses of multiple variates and their interactions for each of ANC05, Platelet20 and Platelet50 detected significant effects in dose group and dose group interactions with diagnosis (Table 3). A close look at regression coefficients of the dose group and diagnosis interaction terms and treatment data suggested the statistical interactions could be triggered by two cases both in the G1 group, with Hodgkin Lymphoma and B‐cell NHL, respectively.

**TABLE 3:** Variance explained and *p*‐values of terms with significant additive or interaction effects in the final regression models.*

	All ASCTs			ASCTs without outliers		
Term	ANC05	Platelet20	Platelet50	ANC05	Platelet20	Platelet50
Sex	0.9% (0.148)	0.2% (0.421)	n/a	n/a	n/a	n/a
Dose group	3.6% (0.036)	4.7% (0.006)	4.5% (0.004)	n/a	2.6% (0.117)	n/a
Diagnosis	2.1% (0.275)	5.0% (0.010)	6.6% (0.001)	5.9% (0.013)	4.6% (0.033)	5.5% (0.016)
Age	n/a	3.2% (0.003)	3.1% (0.002)	n/a	4.1% (0.002)	3.2% (0.008)
Dose group: Diagnosis	9.4% (0.017)	15.9% (1.7e‐05)	21.9% (5.6e‐09)	n/a	7.7% (0.046)	n/a
Sex: Dose group	3.9% (0.028)	3.9% (0.016)	n/a	n/a	n/a	n/a

Abbreviations: ASCT, autologous stem cell transplantation.

* A final regression model was reported for each of the ASCT treatment endpoints ANC05, Platelet20 and Platelet50, in scenarios of using either all ASCT cases or ASCT cases excluding the two identified technical outliers; for each term, ‘Variance explained’ as percentage calculated as the sum square of the term divided by the total variance, p‐value in the bracket was derived from the built‐in F test in the *anova()* function; n/a: not available in the model.

The first patient was a 46‐year‐old female, with primary CNS lymphoma, previously treated with high‐intensity chemotherapy and conditioning regimens (BCNU + Thiotepa). She received a pre‐cryopreservation and post‐thaw CD34 cell dose of 2.5 and 2 × 10^6^ cell/kg, respectively. The time to achieve Platelet20 and Platelet50 was 42 and 69 days. In contrast, five other patients with CNS lymphoma, treated with the same regimen had a shorter median time to achieve these endpoints (i.e., 19 days (range 14–24) for Platelet20 and 22 days (range 14–34) for Platelet50). The second patient was a 60‐year‐old male with Hodgkin lymphoma, treated previously with ABVD and radiotherapy, ChIVPP after the first relapse and ICE after the second relapse. He failed the first mobilization attempt after ICE and G‐CSF and had a second attempt with G‐CSF and plerixafor. After three apheresis procedures, 2.0 × 10^6^ CD34 cells/kg pre‐cryopreservation and 1.7 × 10^6^ CD34 cells/kg post‐thaw were collected. After BEAM conditioning, Platelet20 was reached after 36 days and Platelet50 after 71 days. In contrast, 10 additional patients with Hodgkin lymphoma with higher CD34 dose, had a shorter median time to achieve these endpoints (16 days (range 13–22) for Platelet20 and 19 days (range 16–42) for Platelet50).

**FIGURE 3:**
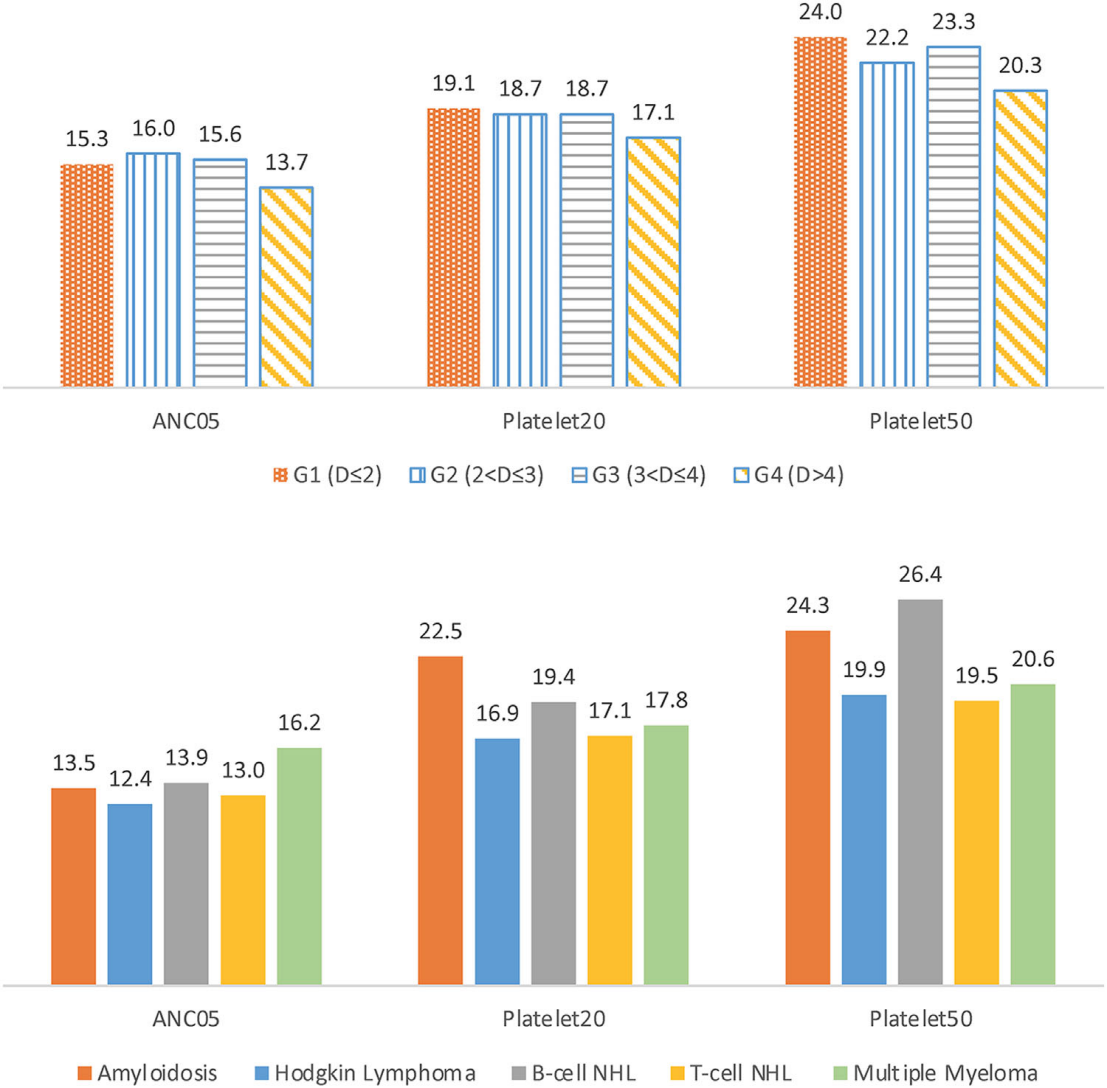
Average days to achieve ANC05, Platelet20 and Platelet50 endpoints post‐exclusion of two technical outliers identified in the low dose group. (A) Cases grouped by dose of CD34 cells (D) where G1 represents the low dose group (D ≤ 2) (top); (B) cases grouped by diagnosis prior to transplant (bottom).

**FIGURE 4:**
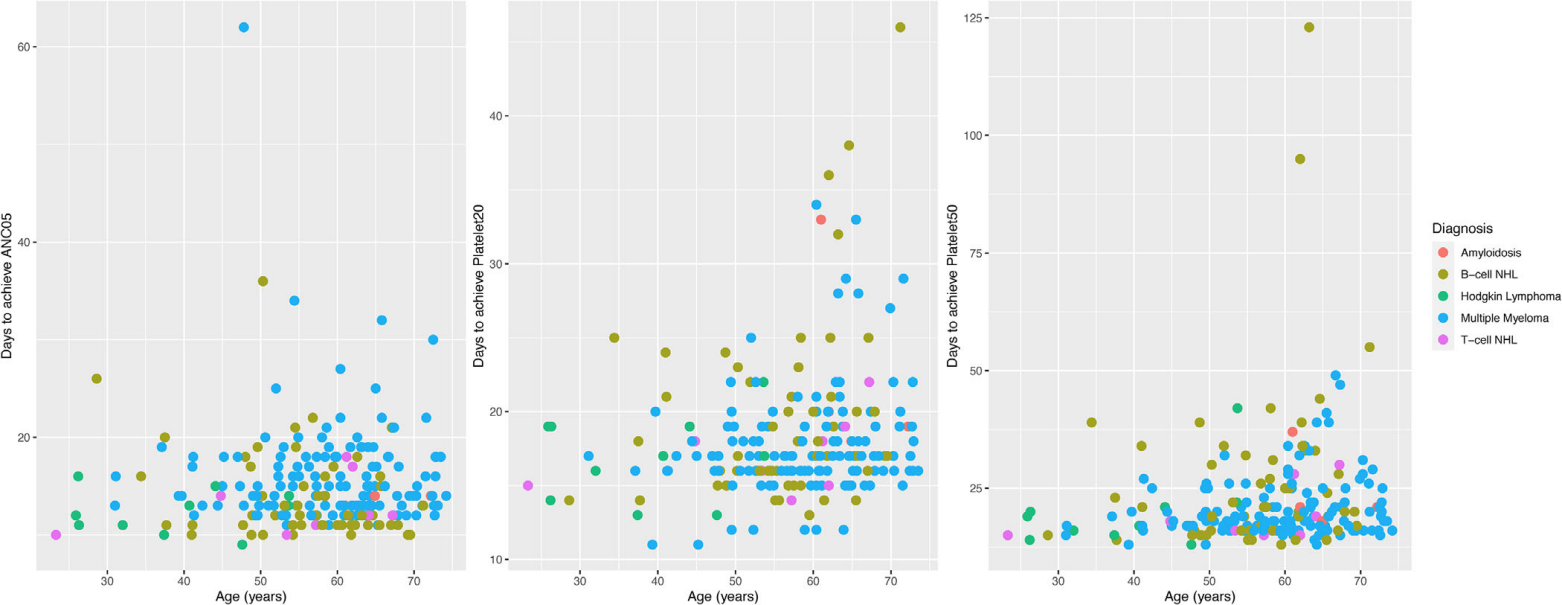
Age effects in achieving ANC05 (left), Platelet20 (middle) and Platelet50 (right) endpoints, post‐exclusion of two technical outliers identified in the low dose group.

After the exclusion of the two outliers, the regression analyses did not find significant effects in either dose group or dose group interactions except for a marginally significant interaction with diagnosis in Platelet20 (Table 3). New plots of the cumulative percentages of cases that achieved each treatment endpoint (Figure 2, bottom panel) and plots of average days to achieve each endpoint (Figure 3, top panel) showed little differences between dose groups. However, significant differences in achieving endpoints between diagnosis groups were observed in ANC05 (*p* = 0.013), Platelet20 (*p* = 0.033) and Platelet50 (*p* = 0.016) (Table 3, Figure 3 bottom panel). In addition, age appeared to be another significant covariate in Platelet20 (*p* = 0.002) and Platelet50 (*p* = 0.008) but not in ANC05 (Table 3, Figure 4). Interestingly, the majority of patients achieved neutrophil and platelet recoveries within 30 days, whereas those with B‐cell NHL took longer to achieve platelet recoveries (Figure 4). The same observation could be made from the plots of cumulative percentages of cases achieved Platelet20 and Platelet50 by the diagnosis group (Figure S1).

## 4 DISCUSSION

In this retrospective study of 217 ASCTs with a total of 347 collections of PBSCs, we critically assessed the relationship of post‐thaw CD34 dose on engraftment in patients with haematological malignancies. We first showed that post‐thaw CD34 was highly strongly correlated with pre‐cryopreservation CD34 and was the second significant predictor explaining ∼2.2% of the total variation of post‐thaw TNC viability—the outcome variable of PBSC processing. After stratifying the ASCT cases into four groups according to the dose of post‐thaw CD34 reinfused, we further showed that for each treatment endpoint, post‐thaw TNC viability was not statistically important and the low dose group was significantly different from the remaining and interacted significantly with the diagnosis group that however were triggered by the outliers. Dose group and associated interactions were no longer significant in general post‐exclusion of the outliers from the same analyses.

Our results can be considered as fresh evidence to support the existing threshold of pre‐cryopreservation 2 × 10^6^ cell/kg CD34s as the minimum dose to initiate ASCT treatment. We could not define the lowest dose necessary for engraftment because all our ASCT cases had pre‐cryopreservation CD34s above the threshold. After excluding two patients who deceased within 2 weeks of cell reinfusion, all the remaining achieved the neutrophil and platelet recovery endpoints (one deceased case even achieved Platelet20 and Platelet50). The post‐thaw CD34 dose was not useful in predicting engraftment outcomes after the exclusion of the two outliers (Table 3, Figures 2 and 3).

Our results can also be considered as an important supplement to the threshold‐based ASCT practices, particularly when patients with pre‐cryopreservation CD34s marginally exceeding the threshold. For such patients, monitoring post‐thaw CD34s and clinical attributes similar to those used in characterizing the two technical outliers could inform better clinical decisions to improve treatment outcomes. Arguably, these technical outliers might tag a small but underexplored space when defining the threshold consensus, that is, there would be a small proportion of ASCT cases with low levels of (either pre‐cryopreservation or post‐thaw) CD34s, who would take longer than expected to recover [1]. We identified them through examining dose group involved interactions that ought not to happen should all dose groups have performed equally. Otherwise, we might have mistakenly concluded that the group with low dose of post‐thaw CD34s would be the worst performer. Nevertheless, international joint efforts are needed to collate sufficient data on of such technical outliers to derive supplemental guidelines for better ASCT practices [7, 19].

Our ASCT cases took longer in neutrophil recovery than those reported in previous studies [7, 9, 12, 13, 20, 21]. One possible reason is that our study concerned only patients with haematological malignancies but without solid tumors and autoimmune disorders. Patients with solid tumors and autoimmune disorders included in previous studies were younger and had higher CD34s and shorter time to neutrophil and platelet recovery, often being submitted to multiple courses of stem cell re‐infusion. Another possible reason is that our institutional protocol did not include the routine use of G‐CSF. It was previously reported that G‐CSF given post‐transplant shortens neutrophil recovery by up to 7 days, although it may not significantly reduce the number of febrile neutropenia days or duration of hospital stay [21, 22, 23]. In addition, our patients did not have blood tests every day, which could potentially prolong some of the time of count recovery and thus make any direct comparison with studies using an inpatient setting and daily blood tests difficult.

Similarly, cross‐study comparisons are unreliable for platelet recovery. For example, Lee et al. [12] reported the outcomes of 36 patients aged 2–70 years old, with haematologic malignancies and solid tumours submitted to ASCT. They found patients with post‐thaw CD34s below 2 × 10^6^ cells/kg took longer to achieve ANC05 and Platelet20 than those with higher CD34 levels. D'Rozario et al. [24] reported in a cohort of 106 ASCT patients aged 17–67 years old, with haematologic neoplasms, solid tumours and autoimmune diseases, that the time to platelet count recovery was significantly shorter in the group with a high post‐thaw CD34 dose (≥3 × 10^6^ cell/kg) than that with a lower dose (<1.5 × 10^6^ cell/kg).

Our results also highlight the disease type as a key indicator of ASCT treatment outcomes measured as time to neutrophil and platelets recovery (Table 3, Figure 3 bottom panel, Figure S1). Our observation of disease type agree with a previous study showing a significant delay in neutrophil recovery associated with the disease type and pre‐transplant status [25]. This may reflect the underlying biology of the diseases treated differentially influencing the stem cell repertoire and/or conditioning regimens. As reported before [7, 25, 26], we detected a significant association of age with both Platelet20 and Platelet50 (Table 3) and speculate the association could be greater in particular disease types such as B‐cell NHL (Figure 4).

This study is limited by a relatively small sample size. We did not study other potentially important outcomes such as longer‐term overall survival, infectious episodes’ rate and transfusion demand.

In summary, in the context of a well‐controlled and standardized Haemotopoietic Progenitor Cell Stability Programme, the pre‐cryopreservation CD34 threshold appears to be the most useful, reliable and convenient marker to predict post‐transplant engraftment. In this scenario, CD34 post‐thaw analysis is not necessary. However, special attention may be needed for some patients with CD34 levels marginally exceeding the threshold (i.e., with a low dose of post‐thaw CD34s re‐infused) who could be clinically complicated and thus require extra time to recover.

## AUTHOR CONTRIBUTIONS

GD, KB and WW conceived the idea of the study. GD, AB, GA, KB and WW contributed to the design of the study and the acquisition, analysis or interpretation of the data. GD and WW performed the statistical analysis and prepared the first draft of the manuscript. AB and KB revised it critically and added important clinical insights. All authors contributed to the content of the paper, and reviewed and approved the final version.

## CONFLICT OF INTEREST STATEMENT

The authors declare no competing interests

## Supporting information

Figure S1: Cumulative percentage of cases achieved ANC05 (left column), Platelet20 (middle column) and Platelet50 (right column) endpoints by diagnosis group. Top panel: concerning all ASCT cases; bottom panel: concerning ASCT cases without two technical outliers identified in the low dose group.
Table S1: Conditioning regimen according to diagnosis.

## Data Availability

The de‐identified data that support the findings of this study are available from the corresponding author upon reasonable request.
