# Supplementary figures and images for: A critical assessment of dose effects of post‐thaw CD34 on autologous stem cell transplantation treatment of haematological malignancies
Source: EJHaem. 2023 Mar 2;4(2):419–27. doi: 10.1002/jha2.665 (PMC10188507; doi:10.1002/jha2.665)

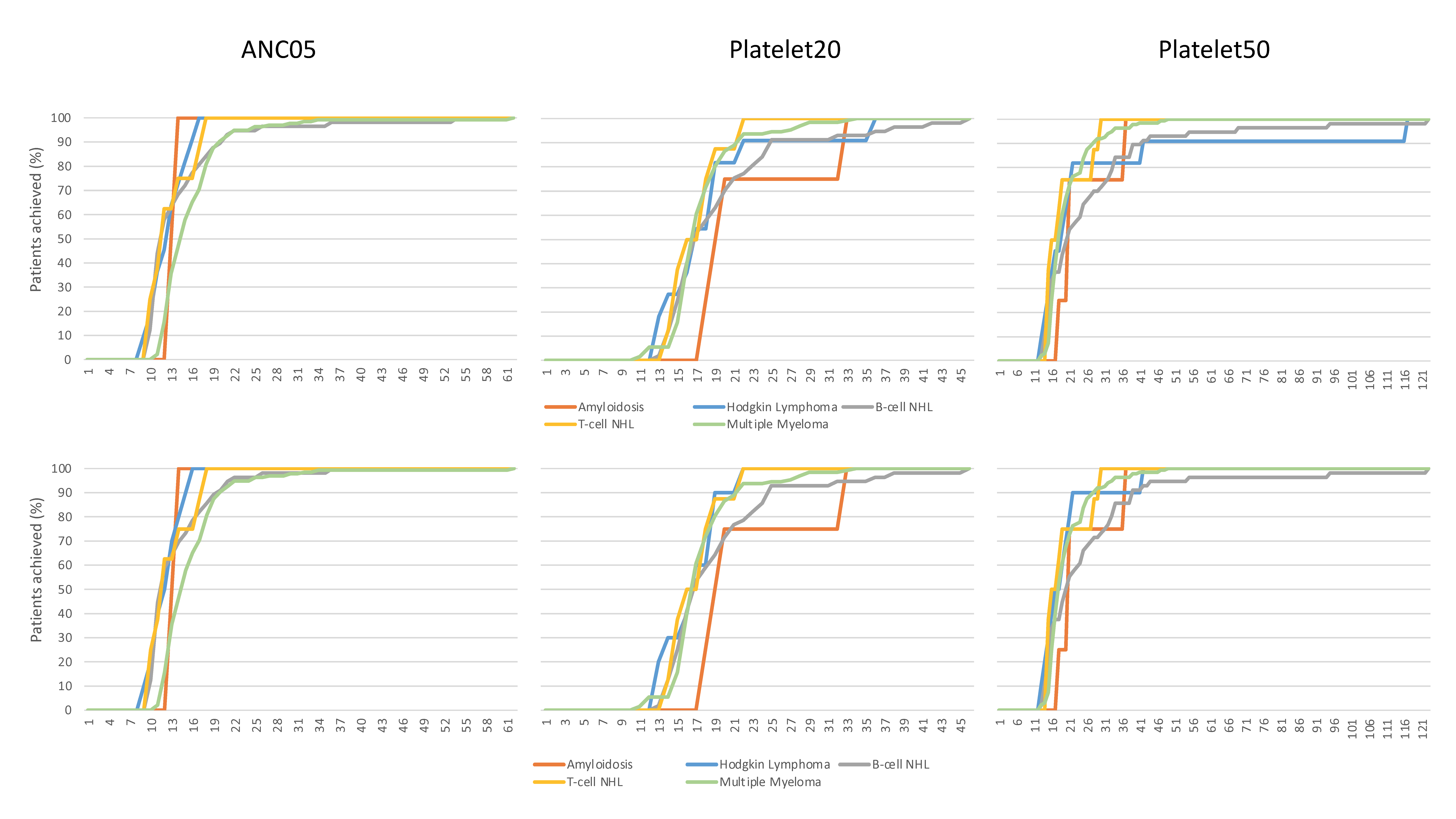

Supplement: Supplementary file 1 — Figure S1: Cumulative percentage of cases achieved ANC05 (left column), Platelet20 (middle column) and Platelet50 (right column) endpoints by diagnosis group. Top panel: concerning all ASCT cases; bottom panel: concerning ASCT cases without two technical outliers identified in the low dose group. Table S1: Conditioning regimen according to diagnosis. [file JHA2-4-419-s001.tiff]
